# From bark to weed: The history of artemisinin

**DOI:** 10.1051/parasite/2011183215

**Published:** 2011-08-15

**Authors:** C. Faurant

**Affiliations:** Consultant, East West Pharmaceuticals 189, rue Grande 77300 Fontainebleau France

**Keywords:** sweet woodworm, malaria, 523 project, artemisinin, ACT, artemisinin combined therapy, history, armoise, paludisme, projet 523, artémisinine, traitement combiné, histoire

## Abstract

In the 1970’s, in China, some brilliant and courageous scientists carried out a research programme, which lead to the discovery of artemisinin derivatives and new quinoleines that are used today, in combination, as first line treatment of malaria.

## Abstract (fr)

Dans les années 1970, de remarquables et courageux scientifiques chinois réalisèrent un programme de recherche qui conduisit à la découverte de dérivés de l’artémisinine et de nouvelles quinoléines qui sont maintenant, en association, des traitements de première ligne du paludisme.

In the midst of time, the Quechuas “doctors” from Peru and Bolivia used the bark of the cinchona tree to produce a medicine to treat some fevers, whereas their counterparts on the other side of the world, in China, used the leaves of sweet wormwood (*Artemisia annua*) for the same effect. The cinchona bark was brought back to Europe in the seventeenth century and empirically used to treat fever and pain until 1820, when the French chemists Pelletier and Caventou isolated the active ingredient, quinine. For centuries to come, quinine has been used to treat malaria successfully, especially after the introduction of its synthetic derivatives at the end of the Second World War (Burns, 2008). One of these derivatives, chloroquine, was dominantly marketed by the company Rhône-Poulenc under the name of Nivaquine®, through two of its affiliates: Specia in the Francophone world and May & Baker in the Anglophone one. Nivaquine® became so popular in Africa that it went beyond being a simple anti-malarial drug and started to be a social phenomenon. The drug was commonly used at low doses to treat any ailments, or just as food supplement. The lyrics of a popular song in DRC (“my Niva*k*ine for Kinshasa”) used the name Nivaquine® to rime with “looking fine” (in French, *bonne mine*). Unfortunately, this fame also signalled the decline of its efficiency as an anti-malarial, which was precipitated by its extensive use during the war between the United States and Vietnam. To remedy this situation, the American army research institute (Walter Reed) launched a programme to find replacement drugs that was at the origin of mefloquine and halofantrine, while the Vietnamese turned to China for help. It is said that Mao Tse-tung himself answered this call and launched the 523 research programme (named after its official starting date, 23rd of May 1967), which not only lead to the discovery of artemisinin but also new quinoleine derivatives that are now used as partner molecules to what is known as “Artemisinin based Combination Therapy” or ACT (*History of Qinghaosu and Prof. Zhou Keding*, 2008).

In 1967, China was at the beginning of the Cultural Revolution, but the scientists chosen to work on the 523 project were promised protection from the Red Guards; nevertheless, considering the speed at which the leaders were replaced then, these scientists (among them, Zhou Keding, the project coordinator in the Academy of Military Medical Sciences, Li Ying from the Shanghai Institute of *Materia medica*...) remained cautious in keeping their work as secret as possible and avoided all communication, which could be intercepted by uncontrollable Red Guards (*History of Qinghaosu and Prof. Zhou Keding*, 2008; Li & Wu, 2003; Burns, 2008). Though later, they liked joking about how they had to work in very special and unorthodox ways, they were taking considerable risks. Recognition was eventually given to them and their remarkable discoveries were acknowledged with the highest State rewards. The secrecy they kept obliged them to hide chemical samples in inconspicuous little flasks that they transported from one place to another to avoid denunciation; all records of their work were written in old books, which could also be easily hidden. It is only later that they could publish their work and progress through better communication (QHS research group, 1980). Unfortunately, it meant that their work could not be conducted properly which explains the difficulties encountered to transform their molecules into medicines and why more work is still needed before some of these compounds can be given safely to patients. If the choice of derivatives and partner drugs, their dosage and ratio have eventually been proved adequate – though not always optimum – the weak points of this programme were definitely the pre-clinical and clinical development. Animal studies were conducted outside all recognised guidelines and cannot guarantee the safety of these products according to modern standards. Moreover, as malaria in China was limited to some remote rural areas with no proper medical structure, the so-called clinical trials were in fact uncontrolled distribution of experimental drugs by health workers, known as barefoot doctors. This will prove to be a serious handicap to the internationalisation of these compounds.

In the early 1970’s, the active ingredient of sweet wormwood was identified as artemisinin (*qinghaosu*) a powerful, though unstable *in vitro* antimalarial. Further studies lead to the development of more stable derivatives, a methyl-ether derivative, artemether and a water-soluble one, artesunate. The synthesis process of these two derivatives needs an intermediate, dihydro-artemisinin – DHA – that was first considered unstable. More recently, it was discovered that both artemether and artesunate were pro-drugs and had to be metabolised into DHA to become active in human blood. Since DHA was then recognised as the active metabolite of all known artemisinin derivatives, further studies (Ashley, 2004) were conducted by the Holley Group to make it more stable and better adapted to the production of a drug. At the same time, new quinoleines were discovered (lumefantrine, pyronaridine, naphthoquine), or re-discovered (piperaquine), and the idea of combining them to the artemisinin derivatives was initiated as early as then.

History changed the course of the 523 project. The relationship between China and Vietnam resumed its usual bitterness; the Americans left a victorious Vietnam, which in turn invaded Cambodia, which lead to a brief Sino-Vietnamese war in 1979. The strategic importance of artemisinin was lost, especially as Vietnamese scientists (Hien, 1994) developed independently an interest in these compounds. The Cultural Revolution was over and the concept of a communist market economy was invented by Deng Xiaoping who encouraged the continuation of the project by independent companies under the supervision of two of his children.

The extraction of artemisinin was assigned to factories of the Holley Group in the Province of Sichuan, where the plant is grown and the formulation of its derivatives into medicines for human consumption was entrusted to two pharmaceutical plants, Kunming Pharmaceuticals for artemether and Guilin Pharmaceuticals for artesunate.

By the beginning of the 1980’s, China started to open up to the rest of the world and the Chinese scientists, including Li Guoqiao (who is at the origin of several ACT), followed the trend (Li *et al.*, 1994). Thanks to Keith Arnold (Arnold, 1993), they contacted professors David Warrel and Nicholas White from the Wellcome Trust and gave them access to data related to the use of artemisinin in the treatment of malaria. These data were first mentioned in a Wellcome Trust publication entitled *A present from Chairman Mao* (Gardner, 2002), which made these compounds known internationally. After this first encounter Nicholas White became a great advocate of artemisinin and through intense lobbying managed to convince the scientific community to take an active interest in these drugs (White *et al.*, 1999). This is also how a first Western company, Rhône- Poulenc Rorer (RPR, now Sanofi-Aventis), decided to study the potential of these drugs and license one of them, injectable artemether, from Kunming Pharmaceuticals. It took four years from the time of the first meeting in September 1989 to the launch in 1993. Negotiations were difficult as the manufacturer could not conduct direct discussions with a foreign company and had to go *via* a state organisation (Citic Group), which did not necessarily follow the same goals. Moreover, the Chinese government suddenly decided to promote its relationship with other emerging countries and all the negotiators found themselves having to pursue their talks in Brazil! Nevertheless, a contract was finally signed in July 1990 in the presence of Deng Zhifang, Deng Xiaoping’s youngest son (Fig. 1). But work only started: the technical dossier was not receivable and had to be totally re-written. New studies were requested, including animal toxicity studies though the product had already been widely used in man! Two major clinical trials were conducted, one in Vietnam (Hien *et al.*, 1996) and one multi-centre in different African countries (Bougnoux & Ancelle, 1993; Danis *et al.*, 1996). Finally an approval for a limited use in French hospitals was granted, which allowed RPR to market this product in endemic areas.

**Fig. 1.**
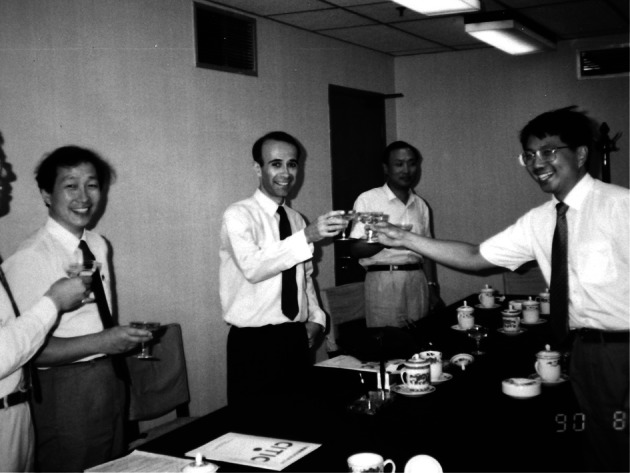
Signature of the contract between Citic (representing Kunming Pharmaceuticals) and Rhône-Poulenc Rorer, in August 1990. From left to right: Li Jiacheng (Project Director in Citic), Claude Faurant (RPR), Cong Zhong (representative of the Ministry of Health), Deng Zhifang (youngest son of Deng Xiaoping).

After this first experience, several Western companies showed an interest in artemisin derivatives, including Novartis, which developed the first ACT artemetherlumefantrine, which is now available worldwide and later Sanofi-Aventis that now produces artesunateamodiaquine for endemic areas. New combinations are also available in endemic areas, but the problem remains how to control these new drugs developed and manufactured outside countries with stringent regulatory authorities (USA, EU, Canada, Australia, New-Zealand...). Moreover, a lucrative market of counterfeit drugs is affecting numerous endemic areas, where regulatory authorities do not always have technical and/or political means to ensure a proper control. In order to palliate this concern, the World Health Organisation – WHO – has recently launched a “pre-qualification” programme enabling companies operating outside countries with stringent regulatory authorities to benefit from the support of international organisations and be able to join international tenders with products of good quality (WHO).

The initial concerns regarding a large usage of ACT were price and availability. The pre-qualification programme has encouraged a Private Public Partnership (PPP) to decrease the price of these drugs in public tenders; a new programme, Affordable Medicines Facility – malaria (AMFm), is under experimentation to decrease the price in the private markets as well. Any pre-qualified products can apply to these programmes, encouraging manufacturing in emerging countries, mainly India and China. The difficulty remains the distribution of the compounds in remote areas, which are the most affected with malaria and where the poorest people live. Another issue is the availability of artemisinin: the concentration of the active ingredient in wormwood is low and vast acres of land are needed to produce sufficient quantities of the Active Pharmaceutical Ingredient (API). Today, only China can provide enough fields to grow plant with high concentration of this active ingredient, though there has been experimental cultivation in several other countries, including Kenya, Tanzania, Madagascar…; new experiments concentrate on increasing the yield of the plant (CNAP), whereas scientists work on synthetic compounds (Medicines for Malaria Venture). But now that China has completely opened up to the world and is an active player in international affairs, partnerships between East and West will work to further improve a remarkable molecule with such an unusual and fascinating history (Table I).

**Table I.** Important dates in the history of artemisinin.

Date	Actors	Action
*circa* 0000	Traditional Chinese Medicine Doctors	Empirical usage of wormwood for fevers
23/05/1967	Chinese government (Mao Tse-toung?)	Launch of the 523 malaria research programme
1971	AMMS*; Shangaï Institute of *Materia medica*	Isolation of artemisinin from wormwood
1972–75	AMMS*; Shangaï Institute of *Materia medica*	Synthesis of artemether, artesunate and DHA
1980’s	Wellcome Trust	Information to the world
1989	Rhone-Poulenc Rorer	Paluther (IM artemether)
1992	Novartis	Coartem (artemether – lumefantrine)
2006	WHO	ACT recommended as first line treatment

* AMMS: Academy of Military Medical Sciences.
